# Endophytic *Bacillus* and *Pseudomonas* spp. Modulate Apple Shoot Growth, Cellular Redox Balance, and Protein Expression Under *in Vitro* Conditions

**DOI:** 10.3389/fpls.2018.00889

**Published:** 2018-06-28

**Authors:** Inga Tamošiūnė, Gražina Stanienė, Perttu Haimi, Vidmantas Stanys, Rytis Rugienius, Danas Baniulis

**Affiliations:** Institute of Horticulture, Lithuanian Research Centre for Agriculture and Forestry, Babtai, Lithuania

**Keywords:** micropropagation, plant stress, plant–endophyte interaction, proteomics, reactive oxygen and nitrogen species

## Abstract

Interactions between host plants and endophytic microorganisms play an important role in plant responses to pathogens and environmental stresses and have potential applications for plant stress management under *in vitro* conditions. We assessed the effect of endophytic bacteria on the growth and proliferation of domestic apple cv. Gala shoots *in vitro*. Further, a model apple cell suspension system was used to examine molecular events and protein expression patterns at an early stage of plant–endophyte interaction. Among the seven strains used in the study, *Bacillus* spp. strains Da_1, Da_4, and Da_5 and the *Pseudomonas fluorescens* strain Ga_1 promoted shoot growth and auxiliary shoot proliferation. In contrast, *Bacillus* sp. strain Oa_4, *P. fluorescens* strain Ga_3 and *P. orientalis* strain G_12 inhibited shoot development. In the cell suspension, the effects of the association between endophytic bacteria and plant cells were specific to each strain. Modulation of the cellular redox balance was monitored in the apple cells using a 2′,7′-dichlorodihydrofluorescein diacetate (H_2_DCFDA) probe, and strain-specific effects were observed that correlated with the *in vitro* shoot development results. Proteomic analysis revealed differences in protein expressions in apple cells co-cultivated with different *Bacillus* spp. strains that had contrasting effects on cellular redox balance and shoot development. The *Bacillus* sp. strain Da_4, which enhanced shoot development and oxidation of H_2_DCFDA, induced differential expression of proteins that are mainly involved in the defense response and regulation of oxidative stress. Meanwhile, treatment with *Bacillus* sp. strain Oa_4 led to strong upregulation of PLAT1, HSC70-1 and several other proteins involved in protein metabolism and cell development. Taken together, the results suggest that different cell signaling and response events at the early stage of the plant–endophyte interaction may be important for strain-dependent regulation of cellular redox balance and development of shoot phenotype.

## Introduction

Plant micropropagation *in vitro* has various applications in germplasm storage, industrial scale production of vegetatively propagated plants, plant biology research, and genetic transformations. However, the application of this method remains limited for recalcitrant plant species or genotypes, and several major agronomic crops still present a challenge (Birch, 1997; Benson, 2000; Pence, 2010). One of the problems with *in vitro* cultivation is that plants are exposed to non-natural conditions, such as synthetic cultivation media, low irradiance, low CO_2_ concentration during light periods, or high air humidity. These factors can lead to an imbalance in the plants physiological equilibria and stress (Benson and Roubelakis-Angelakis, 1994; Cassells and Curry, 2001). The composition of plant growth regulators and/or mineral nutrients into the cultivation medium has been a main focus of studies designed to address the optimization of plant propagation methods *in vitro* (Gaspar et al., 1996; Ramage and Williams, 2002). However, the possible utility of biological interactions with microorganisms to improve plant growth and stress tolerance *in vitro* has rarely been addressed.

In nature, plants live in intimate association with microorganisms that help regulate the plant response to pathogens and environmental stresses (Singh et al., 2011). Endophytic bacteria are a group of endosymbiotic microorganisms that live in plant tissues (Schulz and Boyle, 2006), and the plant growth-promoting properties of endophytic bacteria have been extensively studied (see recent reviews by (Xia et al., 2015; Miliute et al., 2015; Santoyo et al., 2016). In contrast, endophytic bacteria have been often regarded as contaminants of *in vitro* cultures (Kulkarni et al., 2007; Ray et al., 2017). However, several studies have shown that bacterial endophytes are common in plant tissues grown *in vitro* and that their beneficial effects on plant growth indicate that they may be useful as growth-promoting agents. In previous studies, a succession of bacterial communities that colonized pineapple microplant organs *in vitro* were characterized (Abreu-Tarazi et al., 2010) Similarly, endophytic bacteria were isolated from strawberry tissues cultivated *in vitro* (Kukkurainen et al., 2005; Dias et al., 2009), and their beneficial effect on the acclimatization of the seedlings under greenhouse conditions was demonstrated (Dias et al., 2009). Recently, the effects of bacterial endophytes in different *in vitro* culture phases and in different plant organs of *Prunus avium* were studied; isolates of the endophytes *Rhodopseudomonas palustris* and *Microbacterium testaceum* promoted rooting in two difficult-to-propagate genotypes (Quambusch et al., 2016). Botta et al. (2013) demonstrated that *Azospirillum brasilense* and *Gluconacetobacter diazotrophicus* inoculated singularly or together conferred plant growth-promoting activity on tomato plants grown *in vitro*. Further, the drought stress reducing activity of endophytic *Bacillus* and *Pseudomonas* spp. strains was demonstrated in grapevine plants grown *in vitro* (Salomon et al., 2014).

The typical forms of plant–microbe interactions can be categorized into commensal, mutualistic, or pathogenic. However, many microorganisms exhibit different forms of relationships with plants during their life cycles (Newton et al., 2010). It is proposed that at an initial stage, all microorganisms trigger an immune response in plants, while later events lead to the refinement of the interaction based on the capability of the microorganism to escape the host defense response (Zamioudis and Pieterse, 2011; Hardoim et al., 2015). The early events involved in the formation of the plant–microorganism interaction stimulate complex signaling events that include characteristic intracellular accumulation of active oxygen and nitrogen compounds (ROS/RNS), which have also been documented for interactions involving endophytic bacteria (Garcia-Brugger et al., 2006; Bordiec et al., 2011). Although eventually bacterial endophytes settle down as mutualistic colonizers, they may also prime the plant defense reactions and stress tolerance by inducing systemic resistance (Zamioudis and Pieterse, 2011; Pieterse et al., 2014). Previously, bacterial strains of the genera *Bacillus* and *Pseudomonas* have been considered the most common groups to induce systemic resistance (Kloepper and Ryu, 2006). Endophytic microorganisms provide potential means to reduce plant stress under *in vitro* conditions, which would allow for the extension of plant micropropagation techniques into recalcitrant plant genotypes. An outlying objective would be to understand the processes that lead to mutualistic endophyte colonization and the priming of the plant defense and tolerance to stress.

In a previous study, we isolated 38 endophytic bacteria from apple buds of cultivars “Gala,” “Golden Delicious,” and “Orlovim” grown under field conditions (Miliute et al., 2016). Biochemical analysis revealed several traits that are important for plant growth stimulation, including nitrogen fixation and the production of indole-3-acetic acid and siderophores that could have important implications on plant growth under *in vitro* conditions. Therefore, in this study, we assessed the effect of seven endophytic bacteria strains of *Bacillus* and *Pseudomonas* spp. on apple shoot biomass and auxiliary shoot propagation *in vitro*. Further, we used a model system consisting of an apple cell suspension to establish strain-specific effects during the initial stage of plant and microbe interaction, such as microbial cell adherence to plant cells and changes in the cellular redox balance. A proteomic analysis was employed to reveal differences in the expression of proteins participating in the apple cell response to bacterization with *Bacillus* spp. strains, each of which had a unique effect on the cellular redox balance and shoot development *in vitro*.

## Materials and Methods

### Bacterial Strains and Plant Material

Four strains of *Bacillus* spp. and three strains of *Pseudomonas* spp. used in the experiments were isolated from apple buds, as described previously (Miliute et al., 2016). For co-cultivation experiments, the endophytic bacteria were sedimented by centrifugation at the exponential growth phase and resuspended in Murashige-Skoog (MS) medium (Murashige and Skoog, 1962). A final concentration of ∼10^7^ cfu/ml was used for the shoot and cell suspension treatments.

Apple shoots of cv. Gala were maintained on solid MS medium, supplemented with 0.75 mg l^-1^ 6-benzylaminopurine, 30 g l^-1^ sucrose, and 0.8% agar at 25 ± 1°C, under 150 μmol⋅m^-2^⋅s^-1^ intensity illumination for a 16-h photoperiod.

Cell suspensions of cv. Gala were initiated from the callus cell culture that was maintained on Schenk and Hilderbrandt medium (Schenk and Hildebrandt, 1972) supplemented with 9.0 μM 2.4-dichlorophenoxyacetic acid, 0.5 μM kinetin, and 10 g l^-1^ sucrose at 25°C for 4 weeks. Fragments of the callus of approximately 1 g weight were transferred to 25 ml of MS medium supplemented with 9.0 μM 2.4-dichlorophenoxyacetic acid, 4.9 μM indole-3-butyric acid, 0.5 μM kinetin, and 30 g l^-1^ sucrose. The resulting cell suspension was maintained with shaking at 100 rpm at 24°C in the dark. After 2 weeks, the culture was diluted with fresh medium. Cell viability was estimated using Evans Blue dye, as described previously (Baker and Mock, 1994).

### Assessment of the Effect of Endophytes on Shoot Growth and Oxidative Stress Injury

Three microliters of the bacterial suspension in MS medium was inoculated on several nodes of apple shoot leaf petioles that had been transferred to fresh medium 1 day prior to the inoculation. MS medium without bacteria was used for the control treatment. The inoculated shoots were maintained under standard conditions. After 1 week of co-cultivation, oxidative injuries to cellular membranes in the shoot leaf and stem tissues were estimated separately based on quantitative analysis of the lipid peroxidation product MDA, as described previously (Jagendorf and Takabe, 2001). Briefly, the stems and all leaves were removed from 5–6 shoots and were combined into samples of approximately 0.2 g weight, frozen in liquid nitrogen, and homogenized at 26 Hz for 3 min with an MM400 (Retsch Ltd.) homogenizer. To extract the phenolic compounds, the powder was resuspended in 1.5 ml of 50 mM Tris–HCl pH-7.4 containing 1.5% polyvinylpolypyrrolidone and centrifuged at 15000 × *g* for 15 min at 4°C. Five hundred microliters of supernatant were mixed with the same volume of 0.5% 2-thiobarbituric acid in 20% trichloroacetic acid and heated in a boiling water bath for 30 min. After centrifugation at 15000 × *g* for 15 min, the absorbance of the supernatant was measured at wavelengths of 532 and 600 nm, and MDA concentration was estimated using an extinction coefficient of 0.156 μM^-1^ cm^-1^.

Shoot weight and propagation coefficients were assessed after 3 weeks. The shoot weight was designated as the combined weight of the parent and auxiliary shoots. The propagation coefficient was designated as the average number of auxiliary shoots that developed on the parent shoot.

### Analysis of Endophytic Bacterial Association With Plant Cells and the Effect on Cellular Redox Balance

The apple cell suspension was inoculated with endophytic bacteria at a final concentration of ∼10^7^ cfu/ml and cultivated under standard conditions for 6 h. Association of bacteria to plant cells was assessed microscopically, as described previously (Bordiec et al., 2011). The apple cells were sedimented by centrifugation at 600 × *g* for 1 min and associated bacterial cells were quantified using serial dilutions.

Changes to the intracellular redox balance of the apple cells were estimated after 2 and 6 h of co-incubation via H_2_DCFDA staining, as described previously (Joo et al., 2005). Briefly, 20 μL H_2_DCFDA dissolved in DMSO was diluted in 10 ml of MS medium. Cell suspension was dispensed into a 96-well plate and an equal volume of the H_2_DCFDA solution was added (10 μM final concentration). After a 60-min incubation, fluorescence was detected using a fluorometer LS55 (Parkin-Elmer) with Ex = 485 nm, Em = 525 nm and 5 and 2.5 nm slit settings, respectively.

### Apple Cell Proteome Analysis Using Two-Dimensional Electrophoresis

Further, a proteomic analysis of the apple cells co-incubated with *Bacillus* spp. strains Oa_4 and Da_4 was carried out. The cells were treated as described in the previous section and the analysis was performed after a 6-h co-incubation. Samples of apple cell protein were prepared using phenol extraction and ammonium acetate precipitation, as described previously (Isaacson et al., 2006). Four biological repeats were prepared for each treatment. Samples were solubilized in two-dimensional (2D) electrophoresis lysis buffer and protein concentrations were measured using a Bradford assay (Bradford, 1976). Internal standards were prepared from a pooled mixture of all protein extracts.

Protein sample aliquots of 50 μg were labeled with Cy3 and Cy5 fluorescent dyes, and the internal standard was labeled with Cy2 dye (Lumiprobe). After labeling and quenching with 1 mM lysine, protein samples were mixed to include two samples of biological repeats and one internal standard. For the preparative gel, 500 μg of unlabeled internal standard was mixed with 50 μg of labeled internal standard.

Rehydration solution was added to the mixed samples to reach a final volume of 450 μl. Proteins were applied to 24 cm IPG strips with a linear gradient of pH 4–7 and isoelectric focusing was performed with an Ettan IPGphor (GE Healthcare). After the isoelectric focusing, the strips were stored frozen at -20°C. After the two-step equilibration with buffer containing 2% dithiothreitol and then 4% iodoacetamide, the proteins were separated on 1-mm thick 12.5% polyacrylamide gels with Ettan DALTsix electrophoresis apparatus (GE Healthcare). Gels were scanned at 50 μm resolution using a FLA 9000 fluorescence scanner (GE Healthcare). Relative protein quantifications were performed using DeCyder 2-D Differential Analysis Software, v. 7.0 (GE Healthcare).

Preparative gel was fixed in 50% methanol and 10% acetic acid. Protein spots were excised manually and subjected to protein digestion with trypsin, according to a method described previously (Shevchenko et al., 2006). Protein digests were loaded and desalted on a 100 μm × 20 mm Acclaim PepMap C18 trap column and separated on a 75 μm × 150 mm Acclaim PepMap C18 column using an Ultimate3000 RSLC system (Thermo-Scientific), coupled to a Maxis G4 Q-TOF mass spectrometer detector with a Captive Spray nano-electrospray ionization source (Bruker Daltonics). Peptide identification was performed using the MASCOT server (Matrix Science) against *Malus* sp. genome database v.1.0 (Velasco et al., 2010). The threshold value for the identification of proteins was a Mascot score > 50 and at least two peptides.

Blast2GO software (Conesa et al., 2005) was used for the annotation and gene ontology (GO) analysis of the protein sequences identified with the NCBI Protein database. The obtained GO terms were summarized using the REVIGO server (Pesquita et al., 2009) using the *A. thaliana* database and the SimRel semantic similarity method with the level set at 0.7 value. *A. thaliana* homologues of the identified proteins were obtained from the GDR Cyc Pathways Database v. 1.0.2-w^1^ and their interactions were assessed using the String database with default settings (Szklarczyk et al., 2015).

### Statistical Data Analysis

One-way analysis of variance (ANOVA) analysis and Bonferroni *post hoc* test (Prism, GraphPad software Ltd.) were used to establish statistically significant (*p* < 0.05) differences between the means in the measurements of shoot growth, endophytic bacteria association, and H_2_DCFDA assay.

The biological variation analysis module of the DeCyder software was used to match protein spots in four biological repeats across different gels, and ANOVA was used to identify statistically significant (*p* ≤ 0.01) differences in protein abundance. Additionally, a threshold value of at least a 2-fold difference in protein abundance was used.

## Results

### Effects of Endophytic Bacteria on Apple Shoot Growth and Oxidative Stress Injury *in Vitro*

**FIGURE 1:**
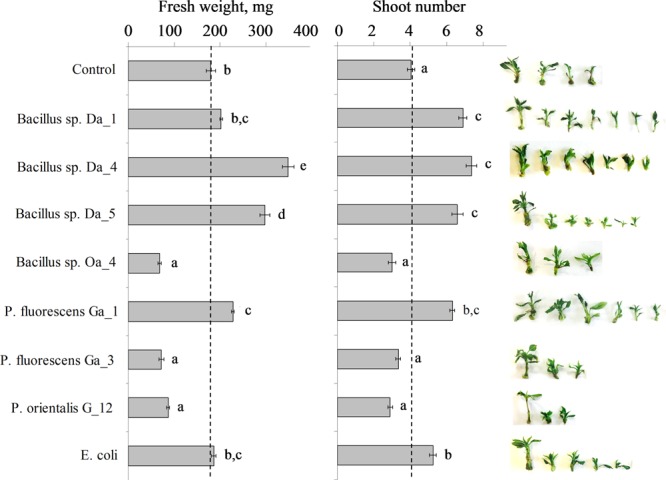
Combined weight of parent and auxiliary apple shoots (left panel) and the auxiliary shoot propagation coefficient (middle panel). Apple shoots were inoculated with 3 μL of each strain suspended at ∼10^7^ cfu/ml in Murashige-Skoog medium. After 3 weeks of co-cultivation, the combined weight of parent and auxiliary shoots and the propagation coefficient represented by an average number of auxiliary shoots that developed on the parent shoot were assessed. A dashed line indicates the mean value of the control shoots. The insert on the right shows a representative sample of auxiliary shoots derived from one parent shoot. Data are presented as mean and SEM of a total of 40–55 shoots from three independent experiments. Means followed by the same letter are not significantly different (*p* < 0.05).

Growth vigor and proliferation of auxiliary shoots are essential parameters for plant propagation *in vitro*. In this study, the effects of endophytic bacteria on the accumulation of biomass and auxiliary shoot proliferation of apple shoots were assessed. Four strains of *Bacillus* spp. and three strains of *Pseudomonas* spp. that had been isolated from apple buds were used in the experiment. After 3 weeks of co-cultivation, observed suppression or stimulation effects varied among strains of the bacterial genera and were distinct to specific bacterial strains. Among the four *Bacillus* spp. strains, Da_4 and Da_5 demonstrated the largest shoot growth enhancing properties. Average FW increased 1.9-fold (349 ± 7 mg) and 1.7-fold (298 ± 11 mg), respectively, compared to the non-treated control shoots (180 ± 10 mg), and the auxiliary shoot number of the apple shoots increased 1.8-fold (average of 7.4 ± 0.3 shoots) and 1.6-fold (6.6 ± 0.3), respectively, compared to an average 4.1 ± 0.2 shoots of the control group (**Figure 1**). The effects of *Bacillus* sp. Da_1 and *P. fluorescens* Ga_1 on the increase in auxiliary shoot number were comparable to those of *Bacillus* spp. Da_4 and Da_5, while *P. fluorescens* Ga_1 led to a significant 1.3-fold increase (228 ± 7 mg) in shoot biomass accumulation compared to control. The bacteria *Escherichia coli*, which was used as a non-endophytic control, had a significant positive effect on the auxiliary shoot formation (average of 5.3 ± 0.2 shoots) as well. In contrast, the remaining three strains (*Bacillus* sp. Oa_4, *P. fluorescens* Ga_3 and *P. orientalis* G_12) suppressed the accumulation of shoot biomass (average FW from 67 ± 7 to 87 ± 2 mg) and the proliferation of auxiliary shoots (average number of shoots from 2.9 ± 0.1 to 3.4 ± 0.2 shoots) (**Figure 1**).

**FIGURE 2:**
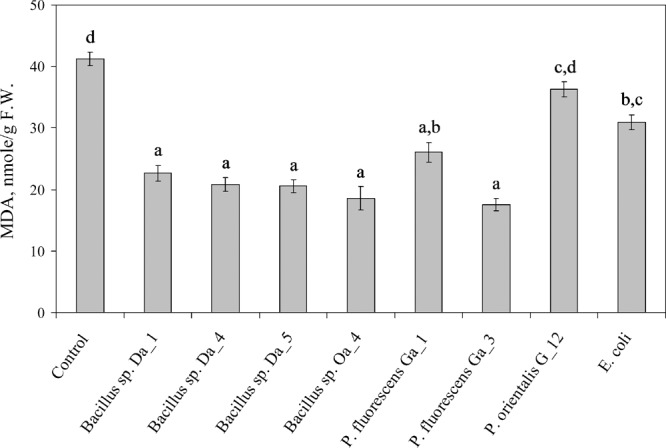
Accumulation of MDA in the leaves of apple shoots co-cultivated with endophytic bacteria. Apple shoots were inoculated with 3 μL of each strain suspended at ∼10^7^ cfu/ml in (MS) medium and observations were made after 1 week. Data are presented as the mean and SEM of at least three repeats from three independent experiments. Means followed by the same letter are not significantly different (*p* < 0.05).

The effects of co-cultivation with endophytic bacteria on oxidative stress injury symptoms induced by cultivation under *in vitro* conditions were assessed separately in the leaf and stem tissues of the apple shoots. In the stems, the concentration of the lipid peroxidation marker MDA remained consistent independent of the bacterial strain used and varied from 9.1 ± 0.2 to 15.9 ± 0.7 nmol/g FW. However, leaf tissues had higher concentrations and statistically significant variations in MDA concentrations (**Figure 2**). Compared to that in control leaves (41.2 ± 1.0 nmol/g FW), MDA accumulation was suppressed 1.6- to 2.3-fold by the majority of the endophytic bacteria strains and its concentration varied from 17.6 ± 1.6 to 26.0 ± 1.0 nmol/g FW. Treatment with *E. coli* led to a significant, but smaller, response compared to other strains (30.9 ± 0.8 nmol/g FW). The exception was *P. fluorescens* G_12, which did not have significant effect on the MDA concentration relative to the control. The higher level of the oxidative stress symptoms was consistent with the negative effect of the strain on shoot growth (**Figure 1**). However, for the other two strains that suppressed shoot growth (*Bacillus* sp. Oa_4 and *P. fluorescens* Ga_3), MDA levels were comparable to that of the strains that stimulated growth and shoot proliferation.

### Interaction of Endophytic Bacteria With Apple Cells in Suspension

A model apple cell suspension system was used to further elucidate the events that occur during the initial phase of endophytic bacteria and plant cell interaction, which may determine subsequent effects on the plant phenotype. We characterized two aspects that had been shown to be characteristic of the early phase of plant host and endophytic or non-endophytic interaction: physical association (Bordiec et al., 2011) and modulation of intracellular ROS accumulation (Nanda et al., 2010).

**FIGURE 3:**
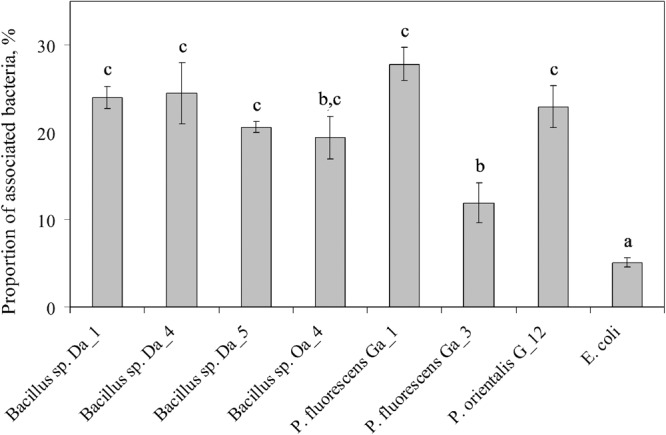
Association of endophytic bacteria with apple cells in suspension. Apple cell suspension was inoculated with each strain at a final concentration of ∼10^7^ cfu/ml. After 6 h of co-incubation, the apple cells were sedimented by centrifugation and associated bacterial cells were quantified by serial dilution. Data are presented as the mean and SEM of at least three repeats from three independent experiments. Means followed by the same letter are not significantly different (*p* < 0.05).

A serial dilution assay was used to estimate the proportion of endophytic bacteria associated to the apple cells after 6 h of co-incubation. As expected, the lowest proportion (5.0 ± 0.5%) of associated cells was observed for *E. coli* (**Figure 3**). For the majority of endophytic bacterial strains, the proportion of associated cells was approximately 4- to 5-fold higher than the *E. coli* control (from 19.4 ± 2.4 to 27.8 ± 3.1%), while the proportion of associated cells for *P. fluorescens* Ga_3 was the lowest at 11.9 ± 1.0%. Although this lower result was consistent with the negative effect of *P. fluorescens* Ga_3 on shoot growth (**Figure 1**), other strains showed association properties independent of their effect on shoot growth. Therefore, it appears that the association of endophytic bacteria with plant cells is universal among the strains of *Bacillus* spp. and *Pseudomonas* spp. and could be an essential property required for endophytic bacterial interaction with plant cells. However, it does not predefine the long-term effect that the bacteria have on the shoot growth *in vitro*.

### Regulation of Intracellular Redox Balance

Due to its complex redox chemistry, H_2_DCFDA is considered inappropriate for the detection of specific ROS, such as H_2_O_2_; however, it is a useful probe for monitoring changes in the redox balance from intracellular sources (Kalyanaraman et al., 2012). The H_2_DCFDA assay was used to detect changes in the redox balance in apple cells after 2 and 6 h of co-incubation with endophytic bacteria. Representative images of cell staining are shown in Supplementary Figure 1 that demonstrate the intracellular accumulation of dichlorofluorescein (DCF) in the apple cells, while no staining of the bacterial cells is detectable.

**FIGURE 4:**
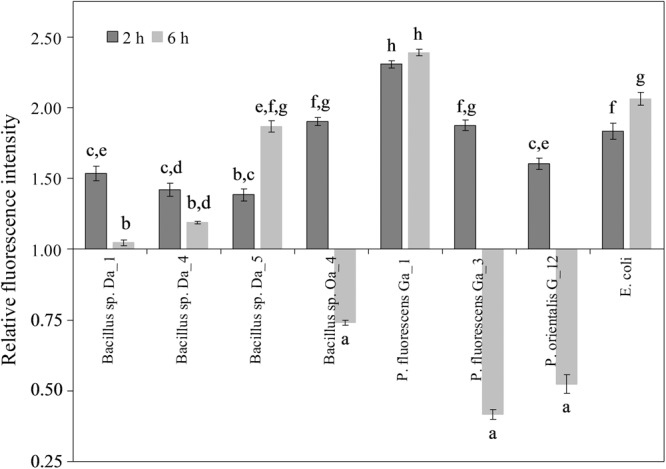
Effect of endophytic bacteria on the accumulation of ROS/RNS in apple cells. Apple cell suspension was inoculated with each strain at the final concentration of ∼10^7^ cfu/ml and observations was made after 2 and 6 h. Data are presented as mean and SEM of at least three repeats from three independent experiments. Means followed by the same letter are not significantly different (*p* < 0.05).

After a 2-h incubation period, the relative fluorescence of DCF was enhanced by all endophytic strains and varied from 1.4- to 2.3-fold compared to untreated cells (**Figure 4**). Similar results were observed after treatment with the non-endophytic *E. coli*. A strain-dependent effect of the endophytic bacteria on the cellular redox balance was observed after 6 h. *Bacillus* sp. Oa_4, *P. fluorescens* Ga_3 and *P. orientalis* G_12 reduced the relative DCF fluorescence staining 1.5- to 2.2-fold relative to the control. The remaining strains either enhanced the fluorescence or did not affect the fluorescence level relative to the control. It is notable that those bacterial strains that reduced DCF accumulation had a negative effect on shoot growth and proliferation, whereas strains that increased DCF accumulation were positively correlated to enhanced shoot growth and proliferation (**Figure 1**).

It is interesting to note that plant cell staining with Evans blue showed that treatment with endophytic bacteria did not affect the viability of apple cells. Cell viability remained between 76 and 86% for all experimental groups, indicating that the endophytic bacterial-induced decrease in DCF fluorescence intensity was not due to a loss of plant cell viability.

### Differential Protein Expression in Apple Cells Incubated With Endophytic Bacteria and Protein Function Analysis

Proteomics analysis was used to assess differential protein expression in apple cells co-incubated with the two endophytic strains of *Bacillus* spp. (Oa_4 and Da_4) that had opposing effects on the intracellular redox balance in the apple cell suspension and on shoot growth *in vitro*. The distribution of proteomes pI values was shown to have a multi-modal character with cytosolic and membrane proteins concentrated mainly in acidic and basic ranges, respectively (Schwartz et al., 2001; Wu et al., 2006). Since a narrow pI range and protein solubility are preferable for efficient protein separation via 2D electrophoresis, the acidic range (pH 4–7) was selected for protein fractionation via isoelectric focusing. The average number of detected protein spots was 1975 ± 197 per gel after alignment. Among the three experimental groups (control and cells co-incubated with *Bacillus* spp. strain Oa_4 or Da_4), 65 proteoforms had statistically significant, >2-fold variations in abundance (**Table 1**). Through liquid chromatography—tandem mass spectrometry (LC-MS/MS) fingerprinting of trypsin digested peptides, 46 proteoforms were unequivocally identified, corresponding to 36 unique *Malus* × *domestica* proteins. One of the proteins was of unknown function and the sequences of the remaining 35 proteins were successfully annotated (data are shown in Supplementary Table 1 and Supplementary Figure 2).

**Table 1:** Number of differentially expressed proteoforms in apple cells co-incubated with *Bacillus* spp. Da_4 or Oa_4 strains.

Differential expression	Oa_4/Control^a^	Da_4/Control^a^	Oa_4/Da_4
Upregulated	32 (29)	10 (7)	30
Downregulated	5 (0)	25 (20)	2
Total	37 (29)	35 (27)	32

*^*a*^Number of proteoforms that had differential expression specific for the strain are indicated in brackets.*

**FIGURE 5:**
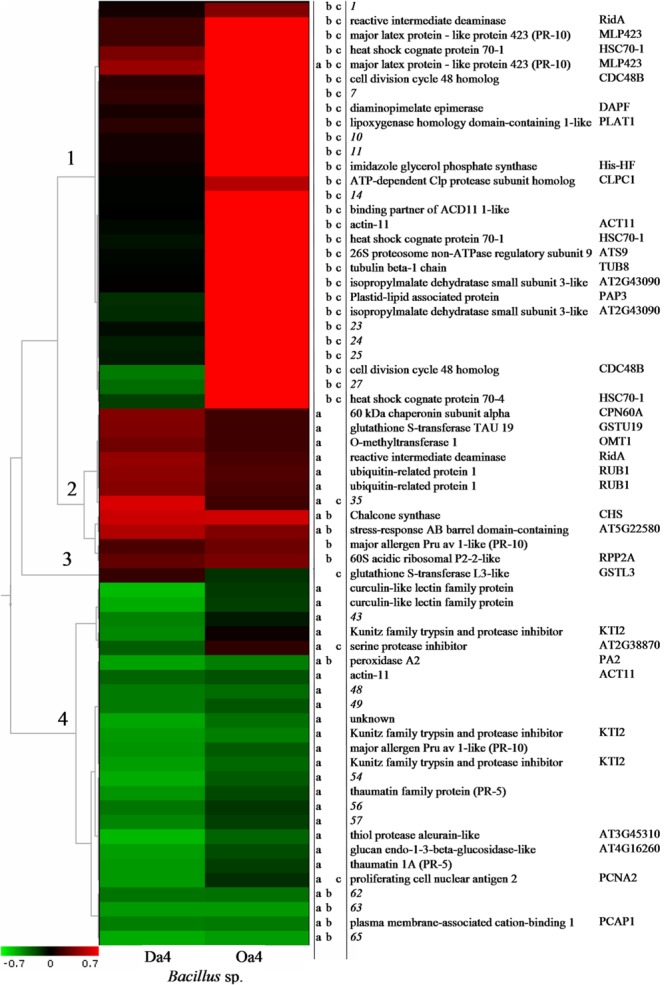
Hierarchical cluster analysis results of the abundance data of proteoforms differentially expressed in apple cells co-incubated with *Bacillus* spp. strains Da_4 or Oa_4 for 6 h. Numbers on the left indicate four major clusters based on expression patterns. Colors indicate a decrease (green) or increase (red) in protein abundance compared to control. Letters in columns 1–3 indicate statistically significant (*p* < 0.01) differences between the Da_4 treatment and control (a), Oa_4 treatment and control (b), and between the Da_4 and Oa_4 treatments (c). Protein names and symbols are shown in columns 3 and 4, respectively.

A hierarchical cluster analysis was used to compare protein abundances of the 65 differentially expressed proteoforms, and four distinct groups were identified (**Figure 5**). The largest group included 28 proteoforms that had large (2.4- to 69-fold) increases in abundance in the apple cells incubated with *Bacillus* sp. Oa_4 relative to control. Among the highly upregulated proteins were lipoxygenase homology domain-containing 1-like protein (PLAT1) (∼69-fold increase) and two of the three differentially expressed proteoforms of heat shock cognate protein 70-1 (HSC70-1) (∼46- and 58-fold, respectively). The abundance of another 19 proteoforms increased more than 10-fold. Meanwhile, only major latex protein-like protein 423 (MLP423) was differentially expressed in samples incubated with the strain Da_4, but the increase was 7.5-fold smaller than the effect of Oa_4. Therefore, the differential expression of proteins that were assigned to this group was specifically induced by the strain Oa_4.

Among the 11 proteoforms included in the second group, the abundance of 9 and 4 proteoforms increased from two- to four-fold after incubation with Da_4 and Oa_4, respectively. Chalcone synthase and stress response AB barrel domain-containing protein (AT5G22580) were upregulated more than 2-fold by both strains. With the exception of reactive intermediate deaminase (RidA) and unidentified proteoform No. 35, another 5 and 2 proteoforms of this group were upregulated less than 2-fold, but a statistically significant difference was still detected after treatment with Oa_4 or Da_4, respectively. Therefore, this group included proteins that had a moderate change in abundance but were responsive to treatment with either of the strains.

In the fourth group, the abundance of 25 proteoforms decreased >2-fold after incubation with Da_4. Peroxidase A2 (PA2), plasma membrane-associated cation-binding protein 1, and three unidentified proteoforms were downregulated by both Da_4 and Oa_4. Although moderate downregulation by Oa_4 was observed for the remaining proteoforms of this group, no significant differences were detected due to the large biological variation. Therefore, it appears that the expression of proteins included in the second and fourth groups had moderate and contrasting responses to treatment with either Da_4 or Oa_4. This might reflect the gene expression patterns that are regulated by these two *Bacillus* spp. or may be a universal response to endophytic bacteria or microorganisms.

Only one protein, GSTL3, was assigned to the third group. Levels of GSTL3 decreased or increased approximately 1.4-fold after incubation with Oa_4 and Da_4, respectively, so that the resulting difference between the two treatments was more than 2-fold.

Further, the analysis aimed to define biological processes associated with the three major groups of differentially expressed proteins. This information should provide hints about the mechanisms involved in the interaction between endophytic bacteria and plant cells that appeared either common or specific for the two strains of *Bacillus* spp. Biological processes were assigned based on GO data, and the String database was employed to assess interactions among the proteins.

The analysis revealed that the first group included the largest number of unique proteins (15) and associated GO terms (37). The latter were summarized based on semantic similarity as eight distinct biological processes (Supplementary Figure 3A), and the dominant processes were related to cell metabolism (56.1%) and protein metabolism (19.0%). In addition, processes related to defense response (11.3%), oxidation-reduction (7.5%), and cell cycle were present (2.5%).

In contrast, nine identified proteins in the second group were associated with the largest diversity of GO terms (33) that were summarized as 11 distinct processes (Supplementary Figure 3B). Biosynthesis was the most dominant (24.4%), and another two processes related to the synthesis of secondary metabolites were present at a lower frequency (0.5%). Processes related to defense response had the same frequency (11.3%) as in the first group. Other processes were related to ribosome biogenesis (2.7%), embryo (1.6%), or chloroplast development (0.8%). It is notable that the uniqueness score of all processes assigned to this group was high (>0.8 for three of the terms and >0.6 for all of the terms), suggesting that the processes are closely related.

The fourth group included 13 identified proteins with 38 GO terms that were summarized to 13 distinctive processes (Supplementary Figure 3C). More than half of the processes were closely related in the semantic space and were associated with various aspects of defense response, such as response to stimulus (12.6%), defense response (6.1%), response to biotic stimulus (5.2%), immune response (1.4%), response to wounding (0.8%), and neutralization of cellular oxidants (including hydrogen peroxide catabolic process) (1.3%). Other processes characteristic to this group included oxidation–reduction (7.5%) and carbohydrate metabolism (4.7%).

**FIGURE 6:**
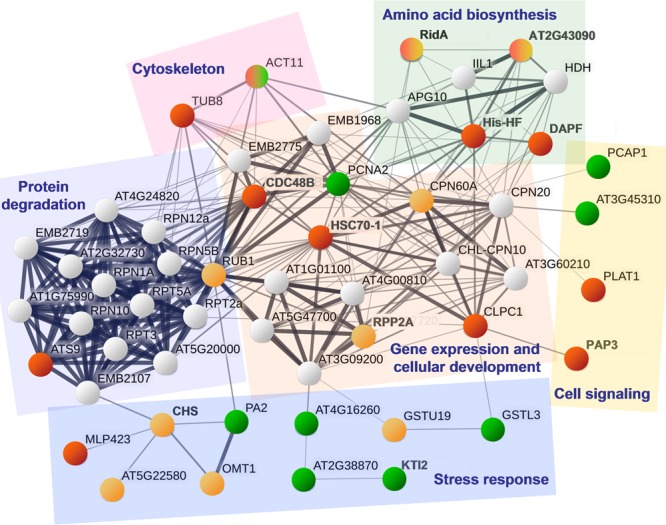
A protein interaction network using *A. thaliana* proteins most closely related to the proteins (groups 1, 2, and 4) that were differentially expressed in apple cells co-incubated with *Bacillus* spp. strains Da_4 or Oa_4. The protein interaction network was built using the String database. Circle colors correspond to the protein group: 1 – red, 2 – orange, 4 – green, and gray circles represent additional network hubs that were not identified in this study. Proteins related by similar functions are outlined with colored shapes.

Because the String database did not include information about apple proteins, 34 unique homologues of *A. thaliana* were used in the analysis of protein interactions. A homologous protein was not available for the apple peptide MDP0000942516 that was annotated as Pru av 1-like protein. Further, no interaction data were available for the RNR-binding motif protein, thaumatin 1A, and curculin-related lectin. Other results revealed associations of the differentially expressed proteins with processes of amino acid biosynthesis and protein anabolism, cytoskeleton, gene expression, and cell development, as well as functions associated with cell signaling and responses to stress, as illustrated in **Figure 6**.

## Discussion

Our study revealed contrasting effects of bacterization with selected endophytic bacteria strains of *Bacillus* and *Pseudomonas* spp. on apple shoot biomass accumulation and proliferation of auxiliary shoots *in vitro*. The stimulating or suppressing effect was not related to bacterial species or genus but varied depending on the specific bacterial strain (**Figure 1**). The stimulating effect on shoot development indicates a mutualistic interaction between the bacteria and plant host; however, the cause of shoot growth suppression is not obvious. Since genotype-specific plant–endophyte interactions have been described for a number of plants (Muller et al., 2015), it could be presumed that the cv. Gala shoots used in the study represented a fully compatible plant host for the strains of *Pseudomonas* spp. which were isolated from buds of the same cultivar grown in the field (Miliute et al., 2016). However, two of the three strains of *Pseudomonas* spp. had negative effects on shoot development. Therefore, it appears that the reduced shoot growth and proliferation could be a consequence of altered interaction between plant host and endophytic bacteria under *in vitro* conditions. This agrees with previous observations that the outcome of the plant–endophyte interaction is determined not only by genetic background and physiological states of the plant host and microorganism but also on environmental conditions (see discussion by Partida-Martinez and Heil, 2011).

As illustrated by the representative samples of auxiliary shoots shown in **Figure 1**, suppressed shoot growth or proliferation was not associated with any adverse effect on shoot morphology, such as distorted morphology or tissue necrosis, that could be symptoms of microbial pathogenesis. In addition, no adverse effect on cell viability was observed during co-cultivation of the endophytic bacteria with the apple cell suspension. A previous study by Bordiec et al. (2011) showed that a challenge of grapevine cell suspension with a pathogenic *Pseudomonas syringae* pv. *pisi* strain induced a hypersensitive-like cell death response. In our study, the apple cell suspension maintained consistent morphology and viability level after a 6-h co-cultivation with endophytic bacteria.

The mutualistic type of interaction between bacteria and apple shoots *in vitro* is further supported by the finding that all bacteria, including the three shoot growth suppressing strains *Bacillus* spp. Oa_4 and *P. fluorescens* Ga_3 and G_12, are able to reduce oxidative stress in the apple shoots (**Figure 2**). This suggests that modulation of oxidative stress by endophytic bacteria might not be the only mechanism responsible for the observed differences in shoot growth. It is likely that the effect is related to bacterial capability to produce substances (e.g., phytohormones) that effect the vigor of plant growth or that the bacteria compete for resources required for plant growth. *Bacillus* and *Pseudomonas* genera include a number of common endophytic bacteria species, and plant growth-promoting traits have been characterized for different strains (see recent reviews by Miliute et al., 2015 and Santoyo et al., 2016). Our previous study established that several of the seven strains showed positive test results for atmospheric nitrogen fixation, nitrate reduction, and production of siderophores or indolyl acetic acid (Miliute et al., 2016). Further, it was established that all seven strains involved in the study were able to maintain growth on minimal medium supplemented with ACC as a consequence of ACC deaminase activity (data not shown). Although direct parallels between these plant growth-promoting traits and the shoot growth-regulating properties established in the present study could not be clearly defined, there is a definite possibility that some of the traits could contribute to the shoot growth regulating effect.

Plant cell suspensions have been used previously to study plant interactions with endophytes (Bordiec et al., 2011; Boonsnongcheep et al., 2016) or rhizobacteria (Verhagen et al., 2010). We used a similar experimental setup to characterize the interaction between the apple cells and endophytic bacterial strains. In the experiments with apple shoots, endophytic bacteria were inoculated at the base of the leaf petiole to perform bacterization of the shoots. Based on the studies carried out *in planta*, it could be presumed that the bacteria would migrate through the shoot tissues over an extended period of time, possibly days (Compant et al., 2005). Such a gradual shoot colonization process would lead to an intricate sequence of perception, signaling, and response formation events in different parts of the shoot. To avoid such complexity, an apple cell suspension was employed as a model system. Although plant cells often form small clumps in the suspension culture, overall it presents a relatively homogeneous population of cells, and co-cultivation of apple cells with endophytic bacteria ensures a uniform response in the cell population. The model revealed distinct morphological, cellular redox balance, and protein expression differences characteristic to the apple and bacterial cell interaction.

Bordiec et al. (2011) demonstrated that the endophytic bacteria *Burkholderia phytofirmans* strain PsJN associates with grapevine cells grown in suspension. Similar results were observed in our study. All endophytic bacteria strains showed a more pronounced association with the apple cells than did the *E. coli* control (**Figure 3**), suggesting that association with plant cells could represent a common characteristic of endophytic bacteria similar to other plant growth-promoting bacteria *in planta* (Rodriguez-Navarro et al., 2007). In contrast, the effect of the 6-h co-cultivation with endophytic bacteria on the intracellular redox balance of the apple cells was strain specific (**Figure 4**). Interestingly, this effect at the early stage of the interaction was correlated with each strain’s capability to regulate shoot growth and proliferation during the 3-week co-cultivation. It was typical that apple cells incubated with shoot growth suppressing strains had reduced levels of DCF fluorescence compared to untreated cells, while fluorescence intensity increased or remained similar to the control in the cells co-cultivated with the shoot growth-promoting strains. Although several distinct processes have been shown to be involved in the oxidation of the H_2_DCFDA probe, several of the one-electron-oxidizing ROS/RNS contribute an important part (Kalyanaraman et al., 2012). Considering this, our results agree with recent findings that showed a capability of endophytic bacteria to regulate ROS production. ROS were produced at early stages of rice root colonization by *G. diazotrophicus* PAL5 *in planta* (Alqueres et al., 2013). Accumulation of ROS/RNS in plant cells bacterized with endophytic strains could be associated with early signaling processes involved in the plant response to microorganisms (Garcia-Brugger et al., 2006) that further leads to activation of systemic resistance (Pieterse et al., 2014).

To further examine biological processes that lead to changes in the cellular redox balance at the early stage of plant–endophyte interaction and that may involve constituents responsible for shoot growth regulation, we assessed changes in the apple cell protein expression induced by the two *Bacillus* spp. strains Da_4 and Oa_4 that had contrasting effects on the cellular redox balance in the apple cells. Four distinct protein expression groups were identified by the cluster analysis of the protein expression data (**Figure 5**) and were associated with similar biological processes involved in metabolism, protein metabolism, defense response, and cell development (Supplementary Figure 3). This suggests that both endophytic bacteria strains elicited responses related to defense and reorganization of cell development. However, very distinct expression patterns of the genes involved in these processes were revealed. The most notable such difference was the upregulation of HSC70-1 and PLAT1 after co-cultivation with *Bacillus* sp. strain Oa_4.

Although the overall function of PLAT1 is poorly understood in plants, one role of the *Arabidopsis PLAT1* gene has been revealed recently (Hyun et al., 2014; Hyun et al., 2015). Overexpression of *AtPLAT1* in *Arabidopsis* and tobacco has confirmed that the gene confers increased abiotic stress tolerance, including cold, drought, and salt stress, and that it promotes plant growth. Analyses of the *AtPLAT1* promoter structure and *in silico* expression data suggest that the gene expression is regulated by ABA and that PLAT1 is a downstream target of the ABA signaling pathway (Hyun et al., 2014). ABA is an important phytohormone that is involved in the regulation of plant water balance and plays a critical role in osmotic and other abiotic stress signaling (Tuteja, 2007). It is known that the ABA response is mediated by ROS accumulation in plant cells (Song et al., 2014). The role of ABA in endophytic bacteria-mediated stress tolerance in plants has been addressed by several studies (Cohen et al., 2009; Salomon et al., 2014; Shahzad et al., 2017). One of the studies performed using grapevine plants grown *in vitro* reported that the drought stress-reducing activity of endophytic strains *Bacillus licheniformis* Rt4M10 and *Pseudomonas fluorescens* Rt6M10 was associated with the accumulation of high ABA levels in the leaves of the bacterized plants (Salomon et al., 2014). In contrast, our results show that upregulation of PLAT1 by *Bacillus* sp. Oa_4 is most likely associated with the suppression of ROS/RNS production in apple cells and that it may also be related to reduced growth and proliferation of apple shoots *in vitro*. This suggests that under *in vitro* conditions, the function PLAT1 does not involve ABA- and ROS-mediated signaling.

HSC70-1, the other protein significantly upregulated after co-cultivation with *Bacillus* sp. strain Oa_4, is homologous to members of the heat shock protein 70 (HSP70) molecular chaperone family. HSPs are involved in the folding of newly synthesized proteins and also play a crucial role in protecting plant cells from the damaging effects of heat stress (Sung and Guy, 2003; Al-Whaibi, 2011). In the *Arabidopsis* genome, there are 14 *HSC70* genes (Cazale et al., 2009). In our study, three differentially expressed proteoforms of HSC70-1 were detected. The proteoforms have different pI value (5, 5.9, and 8.4, respectively) but similar molecular weights (119 kDa) (Supplementary Figure 2) and are arranged in a spot pattern characteristic to the protein post-translational modification that is likely linked to phosphorylation, Therefore, most likely, a single *HSC70-1* gene is upregulated in apple cells by treatment with endophytic bacteria.

Recently, HSPs have received considerable attention due to their function in innate plant immunity (Park and Seo, 2015), while their role in plant–endophyte interactions remains vague. It has been shown that HSP70 accumulates during *Phytophthora infestans*-mediated hypersensitive responses and non-host resistance to *Pseudomonas cichorii* in tobacco (Kanzaki et al., 2003). HSP70 silencing has been associated with increased susceptibility to *Xanthomonas campestris* pv. *vesicatoria* in pepper *Capsicum annuum* (Kim and Hwang, 2015). HSC70-1, together with HSP90 and SGT1, regulates *Arabidopsis* immune responses and is involved in ABA signaling events (Clement et al., 2011). Upregulation of HSPs has been shown in the fungal endophyte colonization of barley roots (Larriba et al., 2015). Downregulation of HSPs induced by non-pathogenic *E. coli* in *Arabidopsis* has also been described (Paungfoo-Lonhienne et al., 2010), but their role in interactions with endophytic bacteria has not yet been defined.

In the present study, the results of String database query revealed a functional network of several proteins closely related to HSC70-1 that were upregulated after treatment with *Bacillus* sp. strain Oa_4 (**Figure 6**). Chaperone-like protein CDC48B has been shown to function in the plant immune response (Rosnoblet et al., 2017), and a protein related to ATP-dependent Clp protease ATP-binding subunit ClpC (CLPC1) has been shown to be involved in the import of proteins into the chloroplast in concert with stromal Hsp93 and Hsp70 chaperones (Flores-Perez et al., 2015). Tubulin 8 (TUB8) and an isoform of actin 11 (ACT11) are components of cytoskeleton. Proteins His-HF, DAPF, and the product of the gene AT2G43090 are enzymes involve in amino acid synthesis. RidA is the enzyme crucial for synthesis of branched-chain amino acids in chloroplasts (Niehaus et al., 2014) and was also shown to function as a N-chlorination-regulated, HSP90-like chaperone in bacteria (Muller et al., 2014), but this function of RidA remains elusive in plants. The described network of functionally related proteins is involved in protein metabolism and cell development. Very prominent changes in protein abundance imply that the treatment with *Bacillus* sp. strain Oa_4 leads to reorganization of the cell development. This could be the same process that leads to the shoot development suppressing activity of this strain.

Treatment with the *Bacillus* sp. strain Da_4 upregulated another proteoform of RidA, chaperone protein CPN60A, and ubiquitin-related RUB1 that are involved in a functional network related to protein expression and cellular development. This could indicate a plant cell response common for the mutualistic interaction with endophytes or at least *Bacillus* sp. bacteria.

In addition, the Da_4 strain led to significant changes in a set of proteins mainly involved in plant defence response or oxidative stress regulation (**Figure 6**). Glutathione-S-transferase (GSTU19) and O-methyl-transferase (OMT1) were upregulated (Gong et al., 2005; Gall et al., 2015). PA2 (Pandey et al., 2017), Ser protease inhibitor (AT2G38870), three proteoforms of Kunitz-type protease inhibitor (KTI2) (Ledoigt et al., 2006; Habib and Fazili, 2007) were downregulated in plants bacterized with Da_4. In addition, not shown in **Figure 6**, several proteoforms of the PR-10 and PR-5 family members, including Pru av 1-like protein and thaumatin 1A (Rajam et al., 2007; Wu et al., 2016) were downregulated. The results imply that downregulation of stress- and defence-related proteins may be important in the less pronounced modulating effect of Da_4 strain on cellular redox balance.

Taken together, our study has established that the endophytic bacteria of *Bacillus* and *Pseudomonas* spp. have a strain-specific capability to regulate apple shoot biomass accumulation and proliferation of auxiliary shoots *in vitro*. This suggests that endophytic biological interaction could help plant explants overcome abiotic stresses encountered *in vitro* and could be useful in the micropropagation of recalcitrant plant genotypes as an alternative to chemical treatment. We show here that molecular events implicated in the early formation stage of the plant host and endophytic bacteria interaction could reflect on the long-term outcome of the interaction and on plant phenotype. Modulation of the cellular redox balance in apple cells during the first hours of interaction could denote a bacterial strain-specific effect on apple shoot development *in vitro*, and the effect has a potential application as a biochemical marker useful for bacterial isolate screening. Further, the proteomic analysis revealed protein expression patterns specific to the strain-specific development of responses in the apple cells. This work provides hints about cell developmental reorganization and stress signaling processes involved in plant host and endophyte interactions under *in vitro* conditions, and it paves the way for further studies on the implicated mechanisms.

## Author Contributions

IT, VS, and DB conceptualized and designed the experiments. IT, GS, PH, RR, and DB acquired, analyzed, and interpreted the data. IT and DB drafted the manuscript. PH, RR, and VS contributed to critical revision of the manuscript.

## Conflict of Interest Statement

The authors declare that the research was conducted in the absence of any commercial or financial relationships that could be construed as a potential conflict of interest.

## Footnotes

1 http://ptools.rosaceae.org/

## Abbreviations

ABA: abscisic acid
ACC: 1-aminocyclopropane-1-carboxylic acid
FW: fresh weight
H_2_DCFDA: 2′,7′-dichlorodihydrofluorescein diacetate
MDA: malondialdehyde
MS: Murashige-Skoog medium
ROS/RNS: reactive oxygen and nitrogen species
