# Supplementary material for: Endophytic Bacillus and Pseudomonas spp. Modulate Apple Shoot Growth, Cellular Redox Balance, and Protein Expression Under in Vitro Conditions
Source: Front Plant Sci. 2018 Jun 28;9:889. doi: 10.3389/fpls.2018.00889 (PMC6032008; doi:10.3389/fpls.2018.00889)
Supplement: Supplementary file 1 [file Table_1.pdf]

Supplementary Table Results of identification and annotation of 65 proteoforms differentially expressed in apple cells co-incubated with *Bacillus* sp. strains Da_4 or Oa_4.

Proteo-form No.	peptide ID at *Malus* sp. genome database	Mascot score	Num. of pep-tides	SC [%]	MW / pI	NCBI-Protein accession ID	TAIR accession ID	Protein name[1]	Protein symbol	Relative abundance[2]		
										Oa4/ control	Da4/ control	Da4/ Oa4
2	MDP0000248778	744	6	40.2	20.7 / 8.3	XP_008365068.1	AT3G20390.1	reactive intermediate deaminase	RidA	13.7	*1.5*	-9.1
3	MDP0000295540	587	6	49.7	17.6 / 5.7	XP_008360260.1	AT1G24020.2	major latex protein - like protein 423 (PR-10)	MLP423	13.8		-90.2
4	MDP0000322220	599	9	10.5	118.9 / 5.9	XP_009374678.1	AT5G02500.1	heat shock cognate protein 70-1	HSC70-1	58.2		-33.4
5	MDP0000750556	450	6	49.7	17.5 / 5.5	XP_008351173.1	AT1G24020.2	major latex protein - like protein 423 (PR-10)	MLP423	19.3	2.6	-7.4
6	MDP0000202700	642	12	18.4	94.1 / 5.2	XP_008359586.1	AT3G53230.1	cell division cycle 48 homolog	CDC48B	11.9		-9.2
8	MDP0000182066	785	13	51.8	29.9 / 5.9	XP_008369843.1	AT3G53580.1	diaminopimelate epimerase	DAPF	8.0		-6.8
9	MDP0000399295	238	3	20.7	20.7 / 5.1	XP_009339735.1	AT4G39730.1	lipoxygenase homology domain-containing 1-like	PLAT1	69.0		-52.2
12	MDP0000879487	804	11	51.9	32.1 / 5.2	XP_008380295.1	AT4G26900.1	imidazole glycerol phosphate synthase	His-HF	7.9		-7.4
13	MDP0000255806	203	6	8.5	119.5 / 6.9	XP_008392716.1	AT5G50920.1	ATP-dependent Clp protease ATP-binding subunit homolog	CLPC1	3.3		-3.3

Proteo-form No.	peptide ID at *Malus* sp. genome database	Mascot score	Num. of pep-tides	SC [%]	MW / pI	NCBI-Protein accession ID	TAIR accession ID	Protein name[1]	Protein symbol	Relative abundance[2]		
										Oa4/ control	Da4/ control	Da4/ Oa4
15	MDP0000243576	510	9	30.3	40.5 / 9.0	XP_008373517.1	AT4G17720.1	binding partner of ACD11 1-like		10.1		-10.5
16	MDP0000183660	1996	17	59.9	41.7 / 5.3	XP_008380137.1	AT3G12110.1	actin-11	ACT11	17.2		-18.5
17	MDP0000322220	524	10	12.3	118.9 / 5.9	XP_009374678.1	AT5G02500.1	heat shock cognate protein 70-1	HSC70-1	46.4		-52.2
18	MDP0000523734	1613	18	52.6	47.2 / 5.9	XP_009356341.1	AT1G29150.1	26S proteosome non-ATPase regulatory subunit 9	ATS9	17.9		-19.9
19	MDP0000487384	1992	21	70.6	50.3 / 4.8	XP_009337361.1	AT5G23860.2	tubulin beta-1 chain	TUB8	6.4		-6.4
20	MDP0000697468	418	4	26.5	26.6 / 6.5	XP_008369444.1 XP_008363792.1	AT2G43090.1	isopropylmalate dehydratase small subunit 3-like		21.8		-22.2
21	MDP0000191668	375	7	40.6	23.3 / 8.7	XP_009349223.1	AT2G35490.1	plastid-lipid associated protein	PAP3	15.5		-21.1
22	MDP0000697468	651	7	42.3	26.6 / 6.5	XP_008369444.1 XP_008363792.1	AT2G43090.1	isopropylmalate dehydratase small subunit 3-like		11.1		-14.3
26	MDP0000202700	483	9	14.3	94.1 / 5.2	XP_008359586.1	AT3G53230.1	cell division cycle 48 homolog	CDC48B	*15.8*		-32.4
28	MDP0000322220	1320	14	17.7	118.9 / 5.9	XP_009374678.1	AT5G02500.1	heat shock cognate protein 70-1	HSC70-1	14.3		-22.5
29	MDP0000614851	1870	16	32.6	62.0 / 5.1	XP_008359517.1	AT2G28000.1	60 kDa chaperonin subunit alpha	CPN60A	*1.5*	2.3	
30	MDP0000122993	1136	17	52.1	25.5 / 5.4	XP_008383009.1	AT1G78380.1	glutathione S-transferase TAU 19	GSTU19	*1.5*	2.2	

Proteo-form No.	peptide ID at *Malus* sp. genome database	Mascot score	Num. of pep-tides	SC [%]	MW / pI	NCBI-Protein accession ID	TAIR accession ID	Protein name[1]	Protein symbol	Relative abundance[2]		
										Oa4/ control	Da4/ control	Da4/ Oa4
31	MDP0000479113	2709	28	79.7	39.8 / 5.8	XP_008347272.1	AT5G54160.1	O-methyltransferase 1	OMT1	*1.5*	2.0	*1.4*
32	MDP0000248778	837	8	50.5	20.7 / 8.3	XP_008365068.1	AT3G20390.1	reactive intermediate deaminase	RidA		2.6	
33	MDP0000141893	157	2	16.2	17.2 / 5.8	XP_008387172.1	AT1G31340.1	ubiquitin-related protein 1	RUB1	*1.7*	2.4	
34	MDP0000141893	279	4	30.5	17.2 / 5.8	XP_008387172.1	AT1G31340.1	ubiquitin-related protein 1	RUB1	*1.6*	2.4	
36	MDP0000208899	2521	18	59.7	43.0 / 5.4	XP_008354921.1	AT5G13930.1	chalcone synthase	CHS	3.6	3.6	
37	MDP0000289300	1525	12	43.8	26.7 / 6.1	XP_008360355.1	AT5G22580.1	stress-response AB barrel domain-containing		2.5	3.1	
38	MDP0000942516	1283	9	68.6	17.5 / 5.6	NP_001281292.1		major allergen Pru av 1-like (PR-10)		2.0	*1.6*	
39	MDP0000323561	522	7	57.0	14.4 / 4.5	XP_008391761.1	AT2G27720.1	60S acidic ribosomal P2-2-like	RPP2A	2.2	*1.9*	
40	MDP0000188674	1545	12	58.0	25.8 / 5.1	XP_008355647.1	AT5G02790.1	glutathione S-transferase L3-like	GSTL3	*-1.4*	*1.4*	2.0
41	MDP0000316310	2679	26	55.6	48.4 / 5.7	XP_008350012.1	AT1G78860.1	curculin-like lectin family protein			-3.2	
42	MDP0000316310	2557	27	57.9	48.4 / 5.7	XP_008350012.1	AT1G78860.1	curculin-like lectin family protein			-3.0	
44	MDP0000318079	238	6	31.3	23.8 / 5.1	XP_008386290.1	AT1G17860.1	Kunitz family trypsin and protease inhibitor	KTI2		-2.4	
45	MDP0000471879	216	5	32.3	17.6 / 6.4	XP_008391169.1	AT2G38870.1	serine protease inhibitor		*1.4*	*-1.8*	-2.5

Proteo-form No.	peptide ID at *Malus* sp. genome database	Mascot score	Num. of pep-tides	SC [%]	MW / pI	NCBI-Protein accession ID	TAIR accession ID	Protein name[1]	Protein symbol	Relative abundance[2]		
										Oa4/ control	Da4/ control	Da4/ Oa4
46	MDP0000448302	637	6	15.9	41.2 / 5.0	XP_008363339.1	AT5G06720.1	peroksidase A2	PA2	-2.1	-2.8	
47	MDP0000157737	259	4	11.1	41.7 / 5.3	XP_008354115.1 XP_008354116.1	AT3G12110.1	aktinas-11	ACT11		-2.0	
50	MDP0000737474	593	7	68.6	16.1 / 5.2						-2.9	
51	MDP0000326576	1993	7	40.7	22.9 / 4.7	XP_008386290.1	AT1G17860.1	Kunitz family trypsin and protease inhibitor	KTI2		-2.7	
52	MDP0000942516	1544	12	77.4	17.5 / 5.6	NP_001281292.1		major allergen Pru av 1-like (PR-10)			-2.6	
53	MDP0000326576	1079	6	40.7	22.9 / 4.7	XP_008386290.1	AT1G17860.1	Kunitz family trypsin and protease inhibitor	KTI2	*-1.8*	-2.8	
55	MDP0000218699	397	6	42.3	25.9 / 4.7	XP_008365751.1	AT1G20030.2	thaumatin family protein (PR-5)	TLP		-2.6	
58	MDP0000764121	1103	9	42.7	38.9 / 6.0	XP_008337854.1	AT3G45310.1	thiol protease aleurain-like			-3.1	
59	MDP0000570395	1296	10	32.2	55.3 / 6.1	XP_008366411.1	AT4G16260.1	glucan endo-1-3-beta-glucosidase-like			-2.8	
60	MDP0000218699	414	3	13.7	25.9 / 4.7	XP_008365751.1	AT1G20030.2	thaumatin family protein (PR-5)	TLP		-2.7	
61	MDP0000153963	719	8	41.9	29.5 / 4.6	XP_008384871.1	AT2G29570.1	proliferating cell nuclear antigen 2	PCNA2		-2.7	-2.1
64	MDP0000201559	1578	15	62.6	23.4 / 5.2	XP_008371940.1 XP_008371938.1	AT4G20260.6	plasma membrane-associated cation-binding 1	PCAP1	-2.1	-2.3	

[1] Name of *A. thaliana* homologue is presented; where *A. thaliana* homologue is not available, protein name assigned in NCBI Protein database is used. [2] Mean values of statistically significant differences of relative abundance between indicated experimental groups; difference value that is lower than 2-fold are indicated in italic. Abbreviations: SC – sequence coverage; MW – molecular weight; pI – isoelectric point.
