# Supplementary material for: Endophytic Bacillus and Pseudomonas spp. Modulate Apple Shoot Growth, Cellular Redox Balance, and Protein Expression Under in Vitro Conditions
Source: Front Plant Sci. 2018 Jun 28;9:889. doi: 10.3389/fpls.2018.00889 (PMC6032008; doi:10.3389/fpls.2018.00889)
Supplement: Supplementary file 2 [file Image_1.pdf]

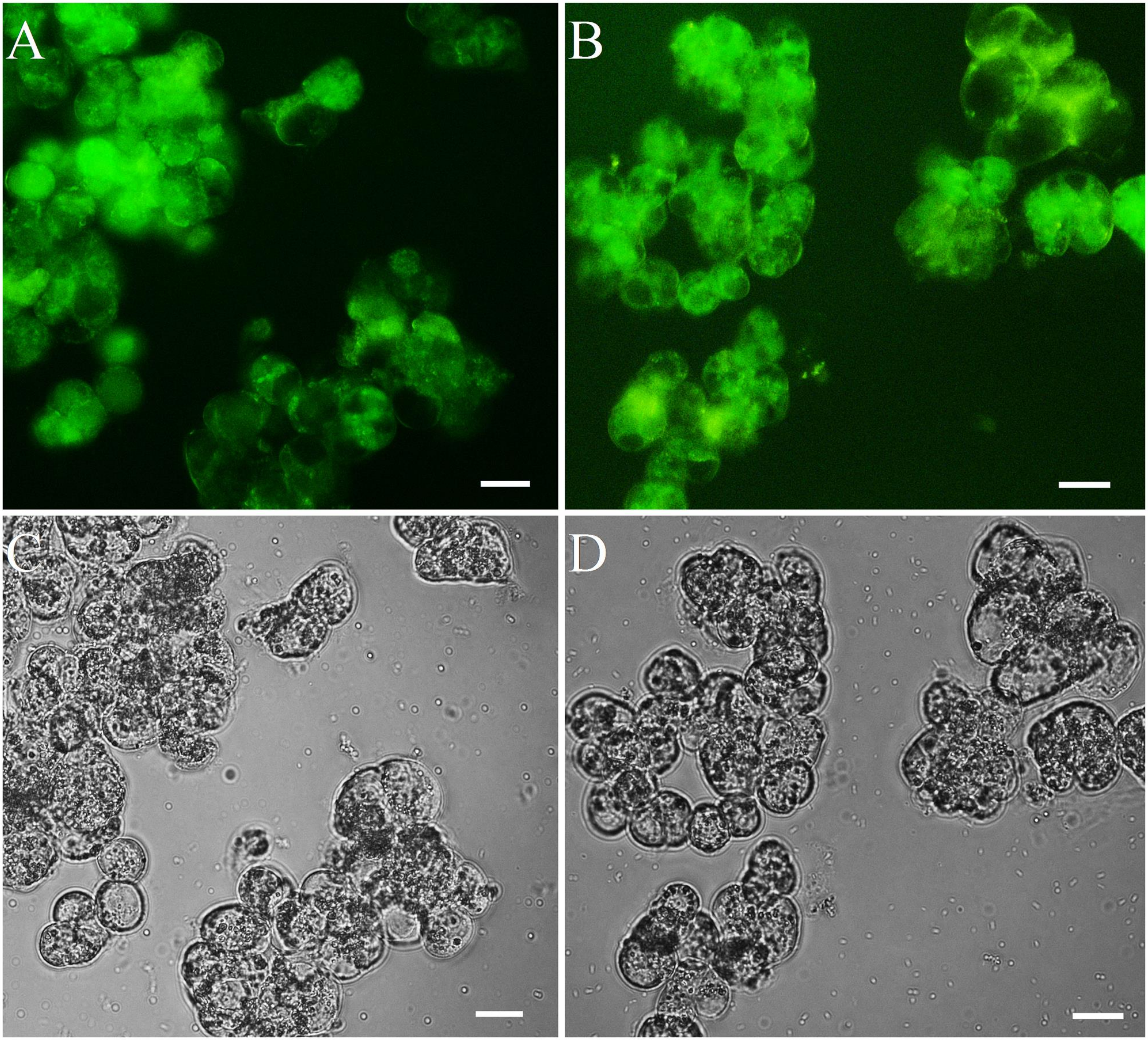

Supplementary figure 1 Representative images of DCF fluorescence (A, B) and bright-field microscopy of the control (A, C) and *Bacillus* sp. Da_4 treated (B, D) apple cells. Scale bar is 10 μm.
