# Supplementary material for: Endophytic Bacillus and Pseudomonas spp. Modulate Apple Shoot Growth, Cellular Redox Balance, and Protein Expression Under in Vitro Conditions
Source: Front Plant Sci. 2018 Jun 28;9:889. doi: 10.3389/fpls.2018.00889 (PMC6032008; doi:10.3389/fpls.2018.00889)
Supplement: Supplementary file 3 [file Image_2.pdf]

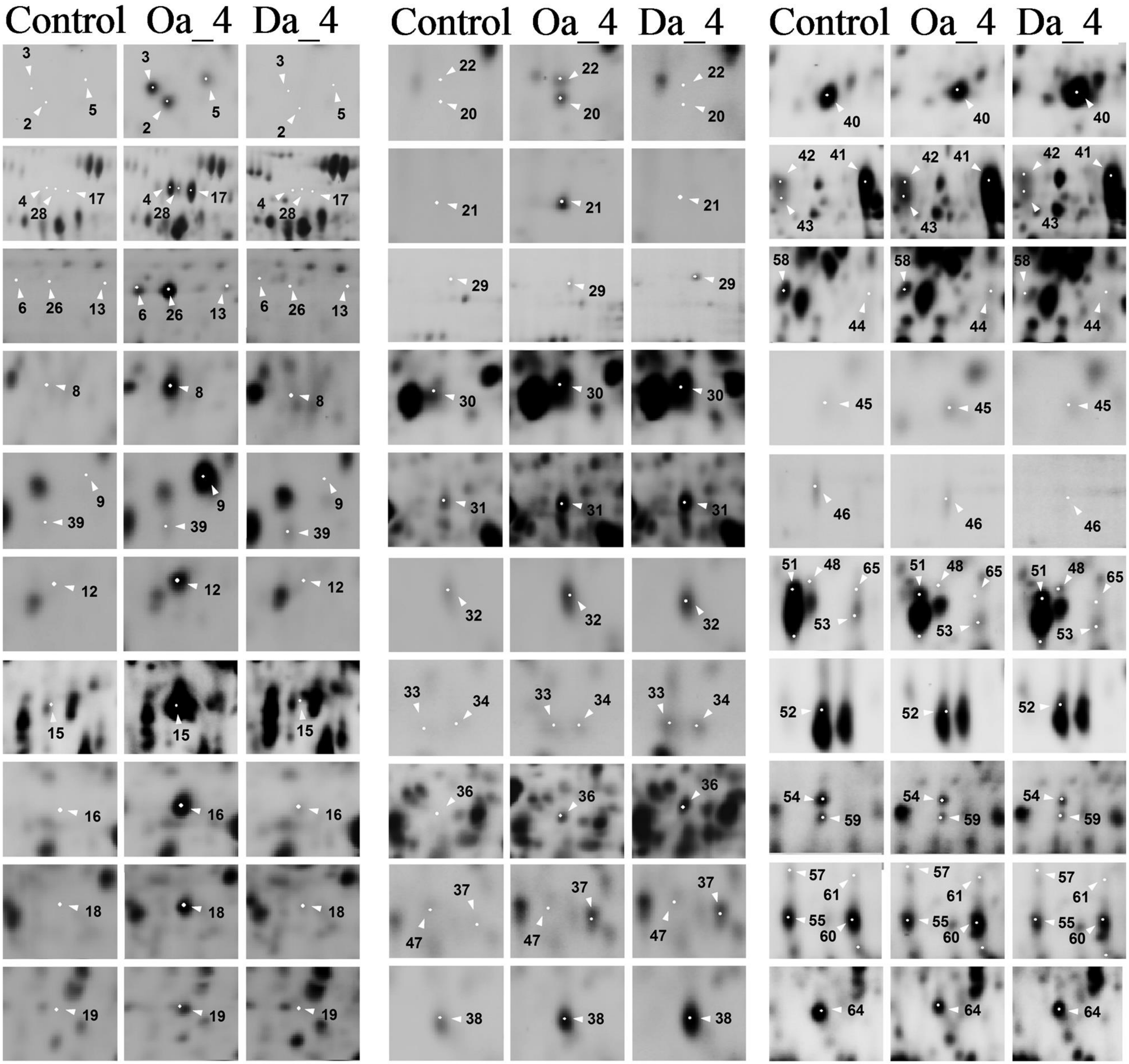

Supplementary figure 2 Gel spots of 46 identified proteoforms differentially expressed in apple cells co-incubated with *Bacillus* sp. strains Oa_4 or Da_4. Representative data of one of the four biological repeats is shown.
