# Supplementary material for: Endophytic Bacillus and Pseudomonas spp. Modulate Apple Shoot Growth, Cellular Redox Balance, and Protein Expression Under in Vitro Conditions
Source: Front Plant Sci. 2018 Jun 28;9:889. doi: 10.3389/fpls.2018.00889 (PMC6032008; doi:10.3389/fpls.2018.00889)
Supplement: Supplementary file 4 [file Image_3.pdf]

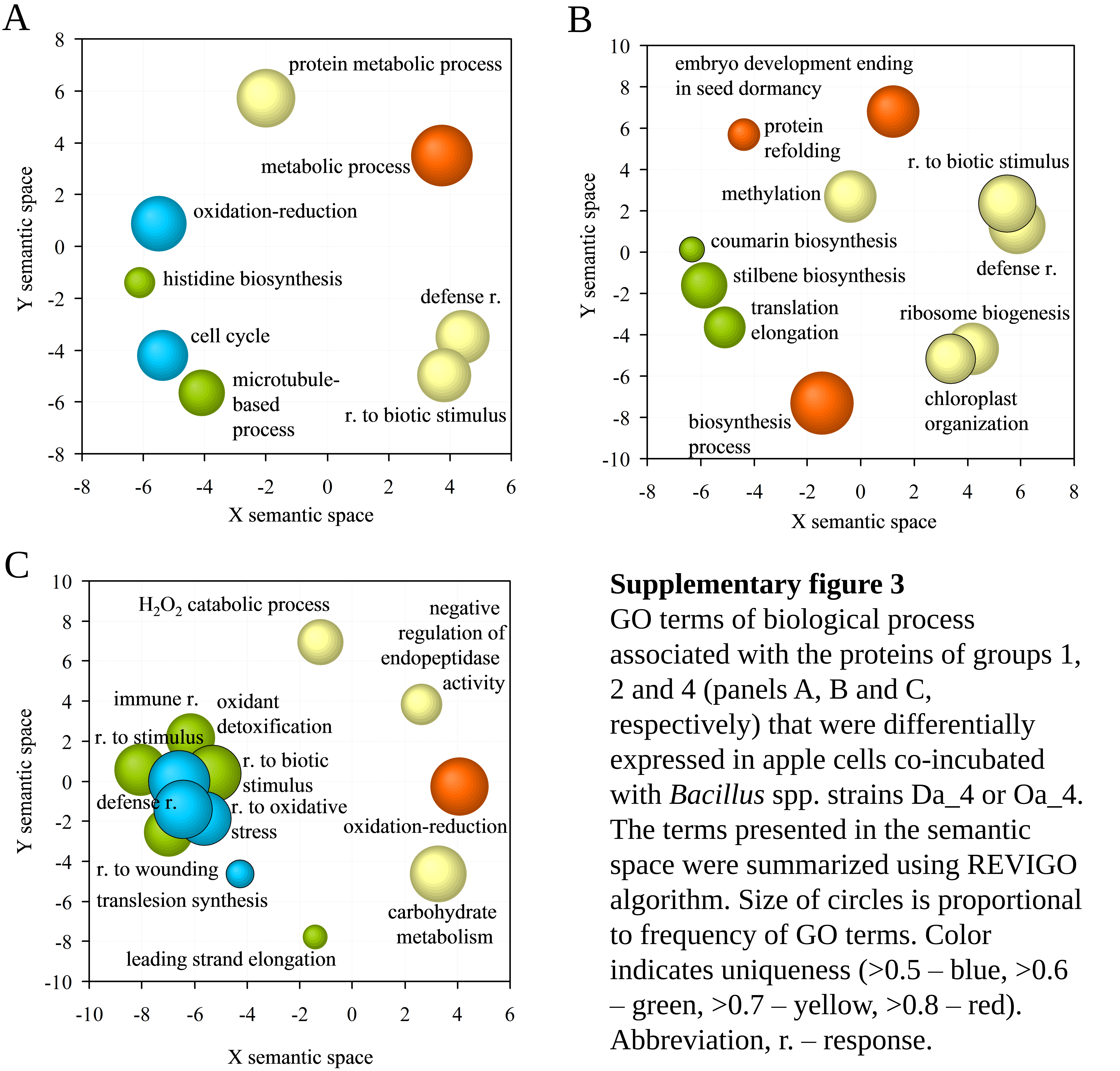

Supplementary figure 3

GO terms of biological process associated with the proteins of groups 1, 2 and 4 (panels A, B and C, respectively) that were differentially expressed in apple cells co-incubated with *Bacillus* spp. strains Da_4 or Oa_4. The terms presented in the semantic space were summarized using REVIGO algorithm. Size of circles is proportional to frequency of GO terms. Color indicates uniqueness (>0.5 – blue, >0.6 – green, >0.7 – yellow, >0.8 – red). Abbreviation, r. – response.
